# MyD88 Deficiency, but Not Gut Microbiota Depletion, Is Sufficient to Modulate the Blood–Brain Barrier Function in the Mediobasal Hypothalamus

**DOI:** 10.1007/s12035-022-02802-w

**Published:** 2022-04-06

**Authors:** Christina N. Heiss, Ellinor Gravert, Matilda Hultén, Louise E. Olofsson

**Affiliations:** Wallenberg Laboratory, Department of Molecular and Clinical Medicine, Institute of Medicine, University of Gothenburg, 41345 Gothenburg, Sweden

**Keywords:** Gut microbiota, MyD88, Blood–brain barrier, Circumventricular organs, Hypothalamus, Tight junction proteins

## Abstract

Circumventricular organs (CVOs), including the mediobasal hypothalamus (MBH), have an incomplete blood–brain barrier (BBB). In this study, we determined if the BBB function in the MBH is modulated by the gut microbiota or by the Toll-like receptor (TLR) adapter proteins TRIF or MyD88 signaling. By injecting mice with Evans blue, a marker for BBB permeability, we show that germ-free (GF) and conventionally raised (CONV-R) mice did not differ in the number of Evans blue-positive cells in MBH. Acute modulation of the gut microbiota did not change the number of Evans blue-positive cells. In contrast, CONV-R *Myd88*^*−/−*^ and *Trif*^*−/−*^ mice had a reduced number of cells in direct contact to the circulation compared to wildtype (WT) mice. This was accompanied by increased tight junction proteins in the blood vessels in *Myd88*^*−/−*^ mice. To further characterize the BBB function, we injected WT and *Myd88 *^*−/−*^ CONV-R mice as well as WT GF mice with monosodium glutamate (MSG), a neurotoxin that does not cross the BBB. While MSG caused vast cell death in the MBH in CONV-R and GF WT mice, *Myd88 *^*−/−*^ mice were protected from such cell death suggesting that fewer cells are exposed to the neurotoxin in the *Myd88 *^*−/−*^ mice. Taken together, our results suggest that MyD88 deficiency, but not gut microbiota depletion, is sufficient to modulate the BBB function in the MBH.

**Supplementary Information:**

The online version contains supplementary material available at 10.1007/s12035-022-02802-w.

## Introduction

The blood–brain barrier (BBB) prevents the entry of potentially harmful blood-derived substances, pathogens, and blood cells into the brain. In the embryo, a primitive BBB is established, which continues to mature after birth [1]. The BBB is composed of highly specialized endothelial cells, which express tight junction proteins including zonula occludens-1 (ZO**-**1), occludin, and claudin-1, -3, and -5. In addition to this endothelial barrier, the BBB also consists of pericytes and astrocytic end-feet, which surround the endothelial cells [1]. Certain areas in the brain, the so-called circumventricular organs (CVOs), have fenestrated capillaries resulting in direct contact between the brain and the circulation, which allows neurons in these areas to sense blood-derived signals or secrete hormones into the circulation. One of these areas is the median eminence in the mediobasal hypothalamus (MBH) [2].

The hypothalamus regulates food intake and energy expenditure, and the neuronal circuits involved in this regulation mature during the first postnatal weeks in rodents. Agouti-related protein (AgRP-) and proopiomelanocortin- (POMC-) expressing neurons are a part of these circuits. They are so-called first-order neurons located in the arcuate nucleus (ARC) in the MBH. The ARC is uniquely located next to the median eminence. Previous studies have shown that ARC also has an incomplete BBB, and cells located in this region can readily sense and respond to substances in the blood that do not cross the BBB [3, 4]. Tanycytes are ependymoglial cells that line the wall of the third ventricle. Previous studies have suggested that tanycytes can regulate the entry of substances into the MBH, potentially via a vascular endothelial growth factor A (VEGF-A)-mediated mechanism [5, 6]. The unique location of the ARC allows neurons located here to sense signals from the body. However, it also makes cells in this region more sensitive to blood-derived molecules. Feeding a Western diet leads for example to an inflammatory response specifically in the MBH [7]. Taken together, the incomplete BBB in the MBH has important consequences for the activation and protection of neurons in this region.

Studies have shown that the gut microbiota can affect the function and development of the CNS including the BBB permeability [8]. A previous study has shown that germ-free (GF) mice have a more permeable BBB compared to conventionally-raised (CONV-R) mice, with reduced expression of tight junction proteins [9]. This study focused on brain areas exhibiting a complete BBB. It is not known if the gut microbiota affects the CVOs differently. Notable, Toll-like receptor 4 (TLR4), that recognizes bacterial and viral products including lipopolysaccharide (LPS), is particularly highly expressed in the CVOs [10–12]. Astrocytes and tanycytes, involved in the BBB function in the MBH, are among the cells that express the receptor in the MBH [10]. Wnt/β-catenin signaling has been shown to be essential for BBB development and maintenance, and the signaling is downregulated in CVOs [13]. Dominant genetic activation of β-catenin in the endothelial cells leads to tightening of the blood vessels in the CVOs [13]. Several studies have shown a crosstalk between Wnt/β-catenin and TLR4/NF-κB signaling [14], suggesting that TLR4 signaling could play a role in the BBB function in these regions. Furthermore, intestinal inflammation has been shown to modulate the brain choroid plexus through bacteria-derived LPS, which in turn regulated Wnt/β-catenin signaling [15]. In this article, we determined if the presence of gut microbes or signaling via the TLR adapter proteins MyD88 and TRIF could modify the BBB function in the MBH.

## Materials and Methods

### Mice

Mice were housed in a room with a 12-h light–dark cycle with free access to water and autoclaved chow diet (Labdiet, St. Louis, MO). Female and male C57BL/6 J mice (10–14 weeks old) were used for immunofluorescence. GF mice were maintained in flexible film isolators and the GF status was monitored regularly by anaerobic culturing and PCR for bacterial 16S ribosomal RNA. All experiments were approved by the ethical committee at the University of Gothenburg. Trif and MyD88 deficient mice have previously been described [16].

### Antibiotic Treatment

Mice were treated with 1 g/L ampicillin and 0.5 g/L neomycin added to the drinking water in light protected bottles for 10 days to 4 weeks. New solutions were prepared every second day. We have previously confirmed that the antibiotic treatment led to depletion of the gut microbiota [7].

### Colonization of GF Mice Using Mouse Donors

Total cecal content from CONV-R donor mice was resuspended in 3 ml of sterile PBS, and each GF mouse was given 200 μl of this solution by an oral gavage. The resulting conventionalized (CONV-D) mice were kept in standard cages for 28 days before used.

### Analysis of the BBB Function Using Evans Blue and Monosodium Glutamate (MSG)

Mice were anesthetized and injected with 1% (wt/vol) Evans blue (Sigma) in 50 µL saline transcardially, and perfused with PBS followed by 4% (wt/vol) paraformaldehyde (PFA) in PBS 10 min later. The brains were dissected, postfixed in 4% PFA, and transferred to 30% (wt/vol) sucrose in PBS overnight at 4 °C. Part of the brain containing the hypothalamus was embedded in OCT (Histolab, Gothenburg, Sweden), frozen and kept in − 80 °C until sectioned in 10 µm thick coronal sections using a cryostat. Direct fluorescence from Evans blue was captured using Zeiss Axioplan 2 imaging system equipped with an AxioCam digital camera HRc using the program Axio Vision 4.8.2.0 (Zeiss, Oberkochen, Germany). At least two different sections were stained per mouse. The positive cells in the MBH (Bregma − 2.15 to − 2.03) were counted blinded and expressed as an average per coronal sections (10 µm thick). Some mice were injected with MSG (0.1 g) or vehicle subcutaneously 24 h before perfusion. MSG solutions were prepared on the day of injection and filter-sterilized under sterile conditions.

### Immunofluorescence

Fed mice were perfused with PBS followed by 4% (wt/vol) PFA in PBS. Brains were dissected and further processed as described above. The brains used for tight junction proteins and MECA-32 staining were only perfused with PBS and transferred to sucrose solution within 4 h. For staining of GFAP and Vimentin, sections containing the MBH were incubated sequentially for 10 min each in 0.3% (wt/vol) glycine solution and 0.3% (wt/vol) SDS solution. For co-staining of GFAP and TLR4 as well as Vimentin and TLR4, no unmasking was used. For HuC/HuD staining, hypothalamic sections were boiled in sodium citrate (10 mM, pH 6) during 10 min using a pressure cooker and then allowed to cool down in room temperature. For staining for MECA32 or for the tight junction protein ZO-1 with Vimentin and/or CD31 slides were fixed in ice-cold 95% EtOH for 30 min, followed by 1 min cold Acetone (histological grade, 534,064, Sigma) and washed in PBS. For claudin-5 together with Vimentin and CD31 staining, slides were fixed for 10 min in 4% (wt/vol) PFA in PBS at room temperature and then washed in PBS. All sections were blocked in 10% donkey or 10% goat serum for 1 h at room temperature, and incubated with primary antibodies (see Table 1) overnight at 4 °C. The sections were then washed in PBS-tween, incubated with respective secondary antibodies (see Table 1) for 1 h at room temperature, and then washed with PBS-tween again. Hoechst solution (dilution 1:10,000, H1399, Thermo Fisher Scientific) was used to visualize cell nuclei. Fluorescence images were captured using Zeiss Axioplan 2 imaging system or Zeiss Imager M1 equipped with an AxioCam digital camera MRm using the program Axio Vision 4.8.2.0 (Zeiss, Oberkochen, Germany) or with a Nikon microscope of the eclipse Ni-E series equipped with an Andor Zyla PLUS sCMOS camera using the program NIS-Element 5.30.04 (Bergman Labora Göteborg, Sweden). The positive cells in the ARC (Bregma − 2.15 to − 2.03) were counted blinded and expressed as an average of at least two coronal sections (10 µm thick). Average tight junction protein immunoreactivity in CD31^+^ blood vessels was measured by Fiji-ImageJ.

**Table 1:** Antibodies. List of primary and secondary antibodies used for immunofluorescence with dilution and catalogue number

Antibody	Dilution	Cat#, company
**Primary antibodies**		
CD31	1:500	NB100-2284, Novus biologicals
Claudin-5	1:500	34–1600, Thermo Fisher Scientific, Waltham, MA
GFAP	1:500	ab53554, Abcam, Cambridge, UK; 13–300, Thermo Fisher Scientific, Waltham, MA
HuC/HuD	1:2000	Ab210554, Abcam
MECA32	1:500	Developmental Studies Hybridoma Bank DSHB
TLR4	1:200	SPC-200, Stress Marq Biosciences
Vimentin	1:1000	AB5733, Sigma Aldrich
ZO-1	1:500	61–7300, Thermo Fisher Scientific, Waltham, MA
**Secondary antibodies**		
AlexaFluor 488 donkey anti-rabbit	1:300	A-21206, Invitrogen, Waltham, MA
AlexaFluor 594 donkey anti-rabbit	1:300	A-21207, Invitrogen, Waltham, MA
AlexaFluor 647 donkey anti-rabbit	1:300	A-31571, Invitrogen, Waltham, MA
AlexaFluor 488 goat anti-chicken	1:300	A11039, Invitrogen, Waltham, MA
AlexaFluor 594 goat anti-chicken	1:300	A11042, Invitrogen, Waltham, MA
AlexaFluor 488 goat anti-rabbit	1:400	A11070, Invitrogen, Waltham, MA
AlexaFluor 594 donkey anti-rat	1:300	A21209, Invitrogen, Waltham, MA
AlexaFluor 488 donkey anti-goat	1:300	A11055, Invitrogen, Waltham, MA

### Cell Death Assay

To determine hypothalamic cell death after vehicle or MSG administration, the In Situ Cell Death Detection Kit (Cat# 11,684,795,910, Merck) was used according to the manufacturer’s recommendations on cryopreserved tissue sections (10 µm).

### Experimental Design and Statistical Tests

Female and male C57BL/6 J mice were used in this study as indicated. Data are presented as mean ± SEM. Each data point in the figures represents data from one mouse. For immunofluorescence analyses, at least two different sections were stained per mouse. Statistical differences were tested with two-sided Mann–Whitney test, or Kruskal–Wallis test with Dunn’s correction for multiple tests. Statistical analysis was performed using GraphPad Prism 9.

## Results

### GF Mice Do Not Have an Altered BBB Function in the MBH

**Fig. 1:**
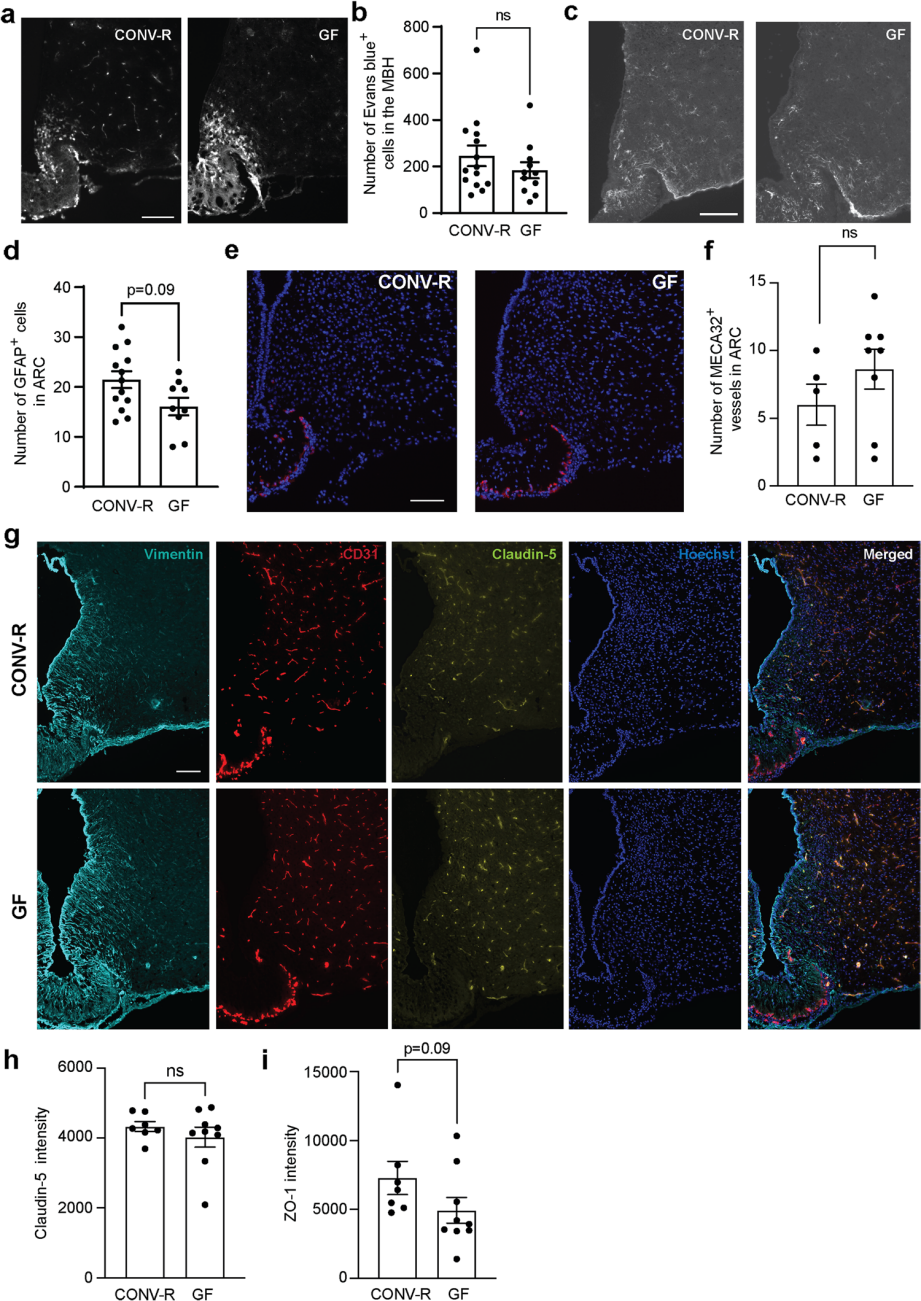
Absence of gut microbiota does not alter the BBB function in the mediobasal hypothalamus. CONV-R (*n* = 14) and GF (*n* = 11) female mice were transcardially injected with Evans blue to mark hypothalamic cells that are in direct contact with the circulation. Sections containing the hypothalamus were analyzed for Evans blue-positive cells (**a**–**b**). *p* = 0.38 as analyzed by a Mann–Whitney test. Hypothalamic sections were stained for the astrocyte marker GFAP and the number of GFAP^+^ cells was determined in CONV-R (*n* = 13) and GF (*n* = 9) female mice (**c-d**). *p* = 0.09 using a Mann–Whitney test. Representative pictures of fenestration marker MECA-32 immunoreactivity in MBH (**e**). The number of MECA-32^+^ blood vessels in ARC (**f**) was determined in CONV-R (*n* = 5) and GF male mice (*n* = 8). *p* = 0.18 using a Mann–Whitney test. Expression patters of vimentin, claudin-5 and ZO-1 were determined in CONV-R (*n* = 7) and GF (*n* = 9) male mice (**g-i**). *p* = 0.68 (claudin-5) and *p* = 0.09 (ZO-1) using a Mann–Whitney test between CONV-R and GF mice. Graphs show mean ± SEM. Scale bars: 100 μm

The gut microbiota has previously been shown to regulate BBB permeability in parts of the brain with a complete BBB [9], and intraperitoneal administration of LPS has been shown to regulate vasculature permeability in the choroid plexus [15]. We hypothesized that the gut microbiota modulates the BBB function in the MBH, a CVO, in healthy mice. To test this hypothesis, GF and CONV-R mice were injected with Evans blue, a marker for BBB permeability, labeling cells outside the BBB in the MBH [3, 4]. The number of Evans blue-positive cells, i.e. the number of cells outside the BBB in the MBH, did not differ between the groups (Fig. 1a-b). We observed a trend of reduced number of astrocytes in the GF compared to the CONV-R female mice (Fig. 1c-d), which is in line with our previous results [7]. There was no difference in the tanycytes (vimentin) or fenestration (MECA-32) pattern in the MBH (Fig. 1e-g). The number of MECA-32^+^ blood vessels in ARC did not differ between CONV-R and GF mice. (Fig. 1e-f), and the tight junction protein expression did not significantly differ between GF and CONV-R mice (Fig. 1g-i). However, there was a trend of reduced ZO-1 immunoreactivity in GF compared to CONV-R mice (Fig. 1i). Taken together, these results suggest that life-long microbiota depletion is not sufficient to alter the BBB function in the MBH.

### Acute Modulation of the Gut Microbiota in Adult Mice Is Not Sufficient to Induce Changes in BBB Function in the MBH

**Fig. 2:**
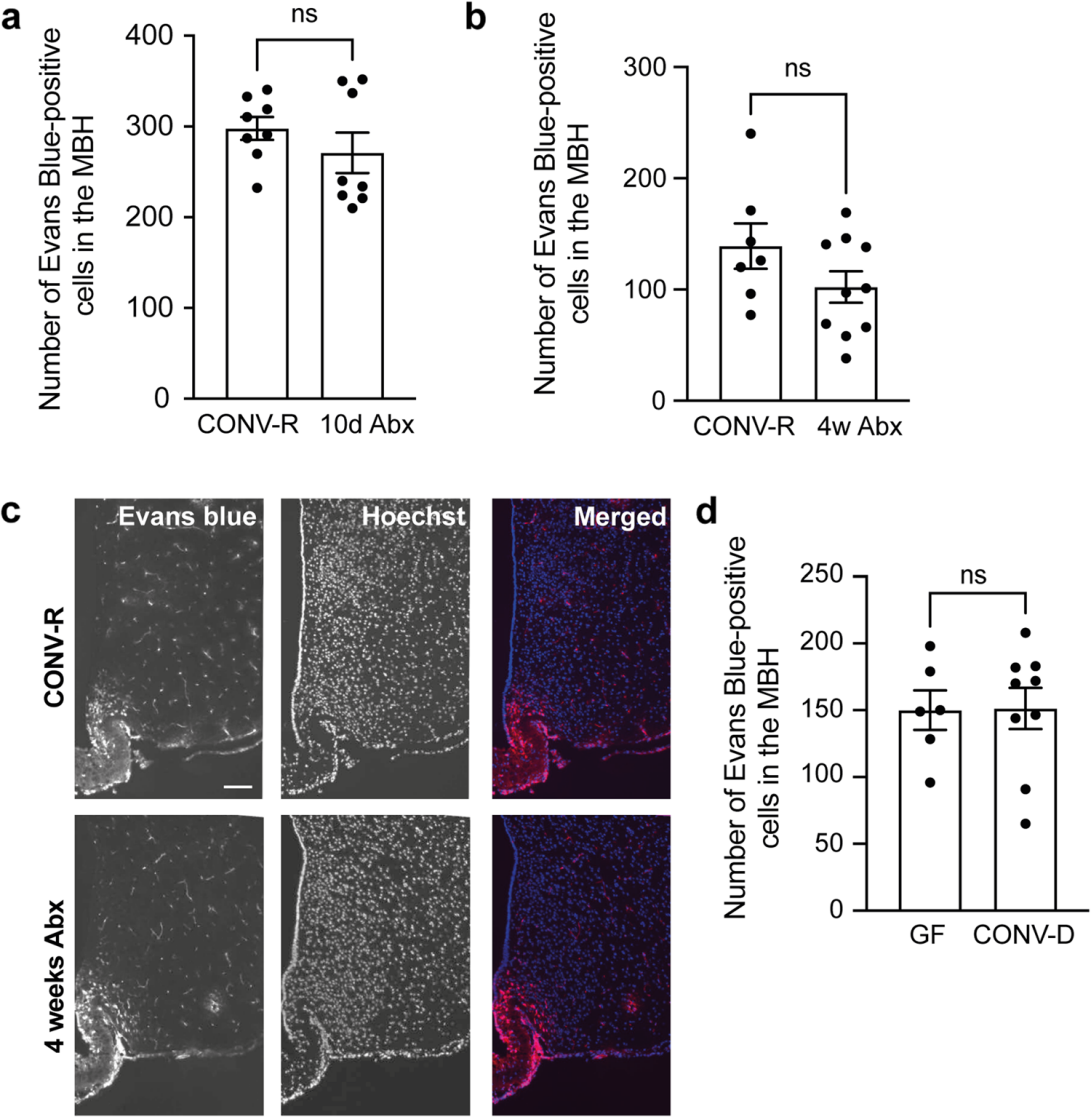
Gut microbiota modulations do not alter the BBB function in the mediobasal hypothalamus. Mice were treated with antibiotics in the drinking water for 10 days or 4 weeks before being injected with Evans blue to mark cells in the hypothalamus that are in direct contact with the circulation. The number of Evans blue-positive cells was determined in CONV-R (*n* = 8) and 10-day antibiotic-treated male mice (*n* = 8) (**a**). *p* = 0.51 as analyzed by a Mann–Whitney test. The number of Evans blue-positive cells was determined in CONV-R (*n* = 7) and 4-week antibiotic-treated female mice (*n* = 10) (**b**–**c**). *p* = 0.23 as analyzed by a Mann–Whitney test. Scale bar: 100 μm. GF mice were colonized by cecal mice microbiota and injected with Evans blue and perfused four weeks later. The number of Evans blue^+^ cells in the mediobasal hypothalamus was determined in GF (*n* = 6) and CONV-D female mice (*n* = 9) (**d**). *p* = 0.95 as analyzed by a Mann–Whitney test. Graphs show mean ± SEM

The lack of difference in BBB function between GF and CONV-R mice may be due to compensatory mechanisms, similar to what is observed in many transgenic mouse models [17]. Therefore, to minimize the impact of compensatory mechanisms, we also determined if the BBB function could be modulated by acutely depleting the gut microbiota from CONV-R mice. We treated CONV-R mice with vehicle or antibiotics for 10 days or 4 weeks and injected Evans blue before perfusing the mice. We have previously confirmed a dramatic depletion of the gut microbiota using this antibiotic treatment protocol [7]. Ten-day or 4-week antibiotic treatment did not alter the number of Evans blue-positive cells significantly (Fig. 2a-c). Similarly, we colonized adult GF mice, and observed no difference in the number of Evans blue-positive cells in the MBH from the colonized mice compared to the GF mice (Fig. 2d). These results suggest that acute modulation of the gut microbiota is not sufficient to affect the number of cells in direct contact with the circulation in the MBH.

### TLR Adapter Protein MyD88 Signaling Modulates BBB Function in the MBH

**Fig. 3:**
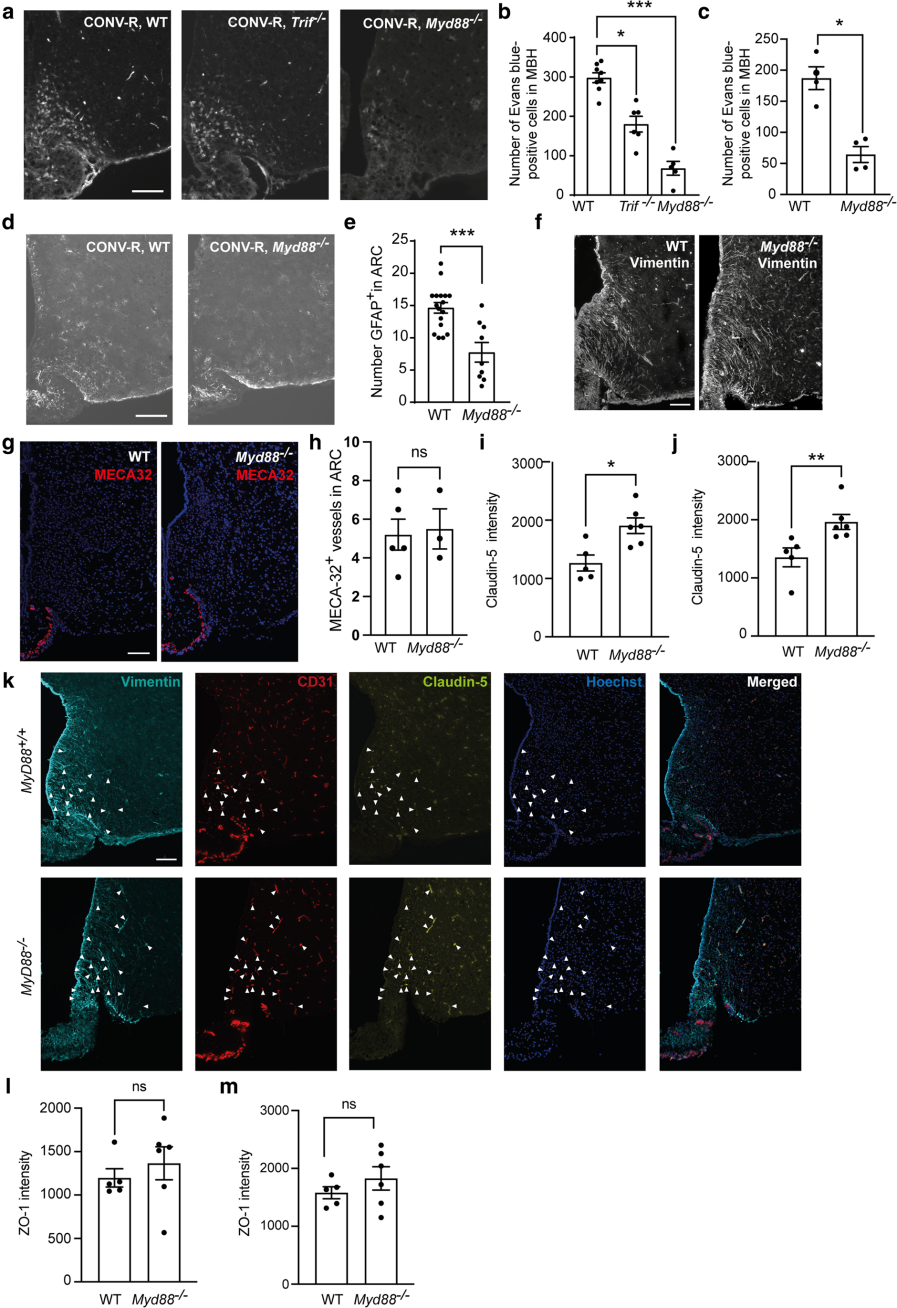
Reduced number of cells in contact with the circulation in mediobasal hypothalamus in *Trif *^*−/−*^ and *Myd88 *^*−/−*^ mice. *Trif *^*−/−*^, *Myd88 *^*−/−*^ and WT mice were transcardially injected with Evans blue to mark hypothalamic cells that are in direct contact with the circulation. Sections containing the hypothalamus were analyzed for Evans blue-positive cells in male (**a**–**b**, *n* = 5–8 per group) and female (**c,** *n* = 4 per group) mice. *p* = 0.047 for male *Trif *^*−/−*^ and WT mice and *p* = 0.0003 for *Myd88 *^*−/−*^ and WT male mice as analyzed by Kruskal–Wallis. *p* = 0.029 for *Myd88*^*−/−*^ and WT female mice as analyzed by a Mann–Whitney test. Hypothalamic sections were stained for the astrocyte marker GFAP and the number of astrocytes was determined in *Myd88*^*−/−*^ (*n* = 9) and WT (*n* = 17) male mice (**d**–**e**). Expression pattern, as analyzed by immunofluorescence, of the tanycyte marker vimentin (**f**) as well as for MECA-32 (**g**), a marker for fenestrated capillaries. *n* = 5 WT mice and *n* = 3 *Myd88 *^*−/−*^ male mice. The number of MECA-32^+^ blood vessels in ARC in *Myd88 *^*−/−*^ and WT mice was counted (**h**). *p* = 0.86 as analyzed with a Mann–Whitney test. Claudin-5 immunoreactivity was determined in blood vessels in ARC (**i**; *p* = 0.017) as well as in blood vessels to which tanycytes connect (**j**–**k**; *p* = 0.0043) in *Myd88*^*−/−*^ (*n* = 6) and WT (*n* = 5) mice. Scale bar: 100 μm. ZO-1 immunoreactivity was determined in blood vessels in ARC (**l**; *p* = 0.66 as analyzed with a Mann–Whitney test) as well as in blood vessels to which tanycytes connect (**m**; *p* = 0.54 as analyzed by a Mann–Whitney test) in *Myd88 *^*−/−*^ (*n* = 6) and WT (*n* = 5) male mice. Graphs show mean ± SEM

Bacteria-derived LPS, a ligand to TLR4, has been shown to modulate the brain choroid plexus through Wnt/β-catenin signaling [15]. Since our results suggest that the absence of gut microbiota is not sufficient to modulate the BBB function in MBH in healthy mice, we next determined if complete deficiency of the TLR adapter protein MyD88 and TRIF signaling could alter the BBB function in the MBH. Previous results suggest that TLR4 is particularly highly expressed in the CVOs of the brain, and that both astrocytes and tanycytes can express the receptor. We confirmed that TLR4 is highly expressed in the MBH and that both astrocytes and tanycytes express the receptor (Supplemental Fig. 1a-e). By injecting mice with Evans blue, we show that adult *Trif *^*−/−*^ and *Myd88 *^*−/−*^ male mice had a reduced number of Evans blue-positive cells in the MBH compared to wild-type (WT) male mice, indicating that signaling via these adapter proteins modulate the BBB function (Fig. 3a-b). Since the difference compared to WT mice was smaller in *Trif *^*−/−*^ compared to *Myd88 *^*−/−*^ mice, we focused on the *Myd88 *^*−/−*^ mice in further experiments. First, we confirmed the reduced number of Evans blue-positive cells in female *Myd88 *^*−/−*^ mice compared to WT female control mice (Fig. 3c). We further examined the number of astrocytes as well as the tanycytes and fenestration patterns. While the number of astrocytes in the ARC was reduced in *Myd88 *^*−/−*^ mice compared to WT control mice (Fig. 3d-e), there was no noticeable difference in the tanycyte or fenestration patterns in the MBH (Fig. 3f-g). We observed a few MECA-32^+^ blood vessels in the ARC in both *Myd88 *^*−/−*^ and WT mice, and the number did not differ between the groups (Fig. 3h). Next, we determined if there were any differences in tight junction protein expression. The claudin-5 immunoreactivity was increased in MyD88 deficient mice compared to WT controls in blood vessels in the ARC as well as in blood vessels which were connected with tanycytic projections (Fig. 3i-k). We did not observe any differences in ZO-1 immunoreactivity in blood vessels in ARC or specifically in the blood vessels to which the tanycytes connect (Fig. 3l-m). Altogether, these results suggest that fewer cells in the MBH directly sense blood-borne substances in mice lacking MyD88 and TRIF, and that increased claudin-5 in blood vessels from MyD88 deficient mice may contribute to this phenotype.

### *Monosodium Glutamate (MSG) Causes Cell Death in CONV-R and GF WT Mice, but to a Lesser Extent in CONV-R Myd88 *^*−/−*^* Mice*

**Fig. 4:**
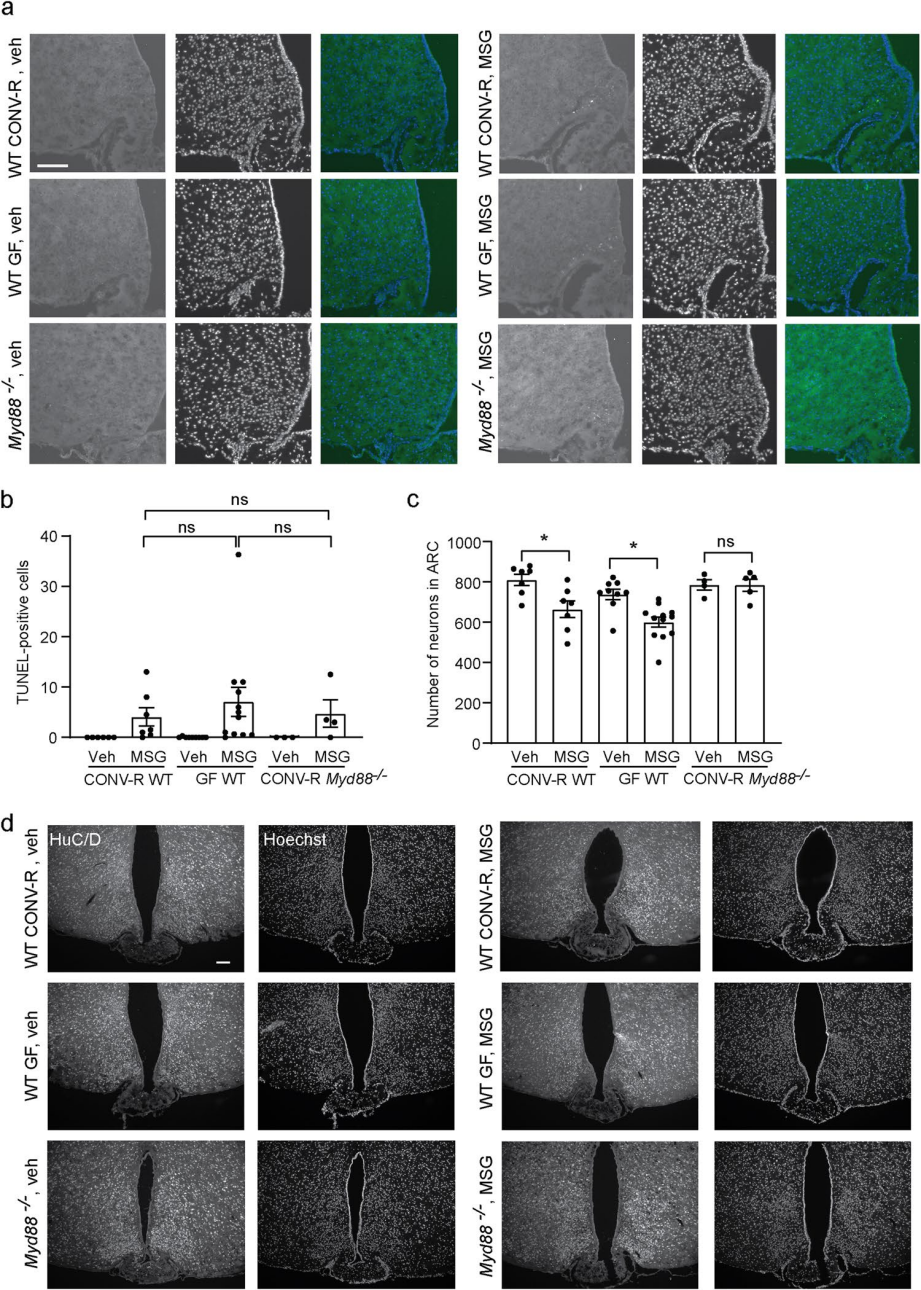
Monosodium glutamate (MSG) causes cell death in CONV-R and GF WT mice, but to a lesser extent in CONV-R *Myd88*^*−/−*^ mice. CONV-R WT, GF WT, and CONV-R *Myd88 *^*−/−*^ female mice were treated with the neurotoxin MSG or vehicle and perfused 24 h later. A TUNEL assay was used to determine cell death in the mice (**a**–**b**). CONV-R vehicle *n* = 6, CONV-R MSG *n* = 7, GF vehicle *n* = 9, GF MSG *n* = 12, *Myd88 *^*−/−*^ vehicle *n* = 3, *Myd88 *^*−/−*^ MSG *n* = 4. *p* > 0.99 for CONV-R WT MSG compared to GF MSG, and *p* > 0.99 for CONV-R WT MSG compared to *Myd88 *^*−/−*^ MSG as analyzed by a Kruskal–Wallis test. Hypothalamic sections were stained for the neuronal marker HuC/D and the number of neurons in the ARC was determined in CONV-R WT, GF WT, and CONV-R MyD88 deficient mice (**c-d**). CONV-R vehicle *n* = 7, CONV-R MSG *n* = 7, GF vehicle *n* = 9, GF MSG *n* = 12, *Myd88*^*−/−*^ vehicle *n* = 4, *Myd88 *^*−/−*^ MSG *n* = 5. *p* = 0.035 for CONV-R WT vehicle compared to MSG, *p* = 0.020 for GF WT vehicle compared to MSG, and *p* > 0.99 for *Myd88 *^*−/−*^ vehicle compared to MSG as analyzed by a Kruskal–Wallis test. Graphs show mean ± SEM. Scale bars: 100 μm

To further functionally characterize the BBB in the MBH, we injected the neurotoxin MSG subcutaneously in *Myd88 *^*−/−*^ and WT CONV-R mice as well as in WT GF mice and perfused the mice 24 h later. MSG is a neurotoxin that kills neurons outside the BBB, but does not cross the BBB [4]. It can therefore be used to assess the BBB function. Two WT CONV-R mice (22%) showed signs of ataxia within a few hours after MSG injection and were therefore euthanized without further analyses. No GF or MyD88 deficient mice had to be euthanized prior to the end of the experiment. To determine ongoing cell death in the MBH, we used a TUNEL-based assay and the number of cells with labeled DNA strand breaks was counted. The number of cells with signs of ongoing cell death did not differ between the three MSG-injected groups using the TUNEL assay (Fig. 4a-b). None of the vehicle-injected mice showed any signs of cell death in the MBH. Since cell death may have occurred prior to the 24-h endpoint, we also determined if the number of neurons was reduced after MSG injection compared to vehicle. While the number of neurons in the ARC was significantly reduced in MSG-injected CONV-R and GF WT mice compared to vehicle, the number of neurons in ARC from MyD88 deficient mice was not decreased compared to vehicle-injected mice (Fig. 4c-d). Furthermore, while cell death was evident by visible inspection of the stained hypothalamic sections in WT GF and CONV-R mice, it was not in the *Myd88 *^*−/−*^ CONV-R mice. These findings support the results from the Evans blue-injections and suggest that fewer cells are in direct contact with the circulation in the MBH, and thereby sensitive to blood-borne substances, in MyD88 deficient mice compared to controls.

## Discussion

**Fig. 5:**
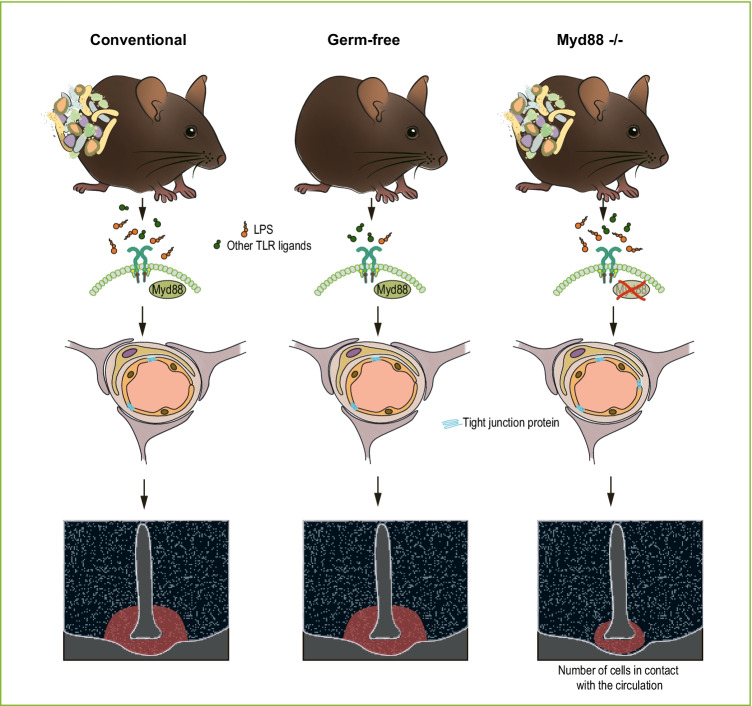
Graphical summary of the main findings of this article. The lack of a microbiota in germ-free (GF) mice does not significantly affect the number of cells in contact with the circulation in the MBH compared to conventional mice. The expression of tight junction proteins in the blood vessels in the ARC did not differ between CONV-R and GF mice. In contrast to the GF mice, depletion of the Toll-like receptor adapter protein Myd88 resulted in increased expression of tight junction protein claudin-5 as well as reduced number of cells in contact with the circulation in the MBH

In this study, we show that TLR adapter proteins MyD88 and TRIF deficiency, but not gut microbiota depletion, are sufficient to modulate the BBB function in the MBH (Fig. 5). While MyD88 and TRIF deficient mice had a reduced number of cells in direct contact with the circulation compared to WT CONV-R mice, GF WT mice did not differ from CONV-R WT mice. Furthermore, we show that MyD88 deficient mice are less sensitive to circulating toxins. Subcutaneous administration of MSG caused extensive cell death in the mediobasal hypothalamus in GF and CONV-R WT mice, but not in MyD88 deficient mice.

MyD88 is central in TLR/interleukin-1 receptor (IL-1R) signaling, and all TLRs/IL-1Rs except TLR3 utilize MyD88 [18]. Thus, the phenotype we observed in MyD88 deficient mice may be due not only to diminished TLR4 signaling but also to other TLRs and IL-1Rs signaling. In contrast to the wide usage of MyD88 in TLR/IL-1R signaling, only TLR3 and TLR4 utilize TRIF. We observe a similar but less pronounced phenotype in TRIF deficient mice compared to the MyD88 deficient mice, suggesting that at least some of the effect is due to TLR4 signaling.

TLRs, including TLR4 and TLR2, are particularly highly expressed in CVOs indicating an important role of these receptors in detecting the presence of invading microorganisms in these regions [10–12, 19]. Notable, TLR signaling is tightly regulated by negative feedback to avoid detrimental effects, and MyD88 can act as an intracellular negative regulator to protect against TLR overactivation [20]. Laflamme et al. found that LPS administration diminished the expression of TLR4 in the CVOs including the mediobasal hypothalamus [12]. Together with our results, these results suggest that the decreased TLR4 expression may act as a protective mechanism leading to increased tight junction protein expression during systemic infection, which in turn inhibits microorganisms to enter the CNS via CVOs.

TLR4 recognizes bacterial and viral products including LPS [21], but it has also been suggested that TLR4 signaling can be activated and modulated by endogenous signals and dietary fatty acids [22, 23]. These dietary fatty acids and endogenous ligands can potentially activate TLR4 signaling in GF mice. Furthermore, grain-based rodent diets, which has been used in this study, have previously been shown to contain bacterial parts and components including LPS and that such components can affect experimental results using GF mice [24, 25]. Taken together, TLR4 may also be activated in the absence of microorganisms and complete deletion of the adapter proteins in the knockout mice may therefore cause a more sever phenotype, as observed in this study.

Previous studies have shown that LPS induce tight junction permeability in the gut via TLR4-induced activation of membrane-associated adaptor protein FAK and MyD88, and silencing of MyD88 using siRNA prevented LPS-induced tight junction permeability [26]. LPS administration in mice have also been shown to increase BBB permeability, an effect that was associated with increased MyD88 gene expression and decreased tight junction expression in the brain [27]. In an in vitro model of brain microvascular endothelial cells, LPS exposure led to an altered morphology and staining patterns for ZO-1 and claudin-5 [28]. Together with our study, these studies suggest that LPS-TLR4-MyD88 signaling can regulate BBB function by altering the tight junctions.

Wnt/β-catenin signaling regulates BBB development and maintenance in endothelial cells in CNS vessels during embryonic and postnatal development [29]. Benz et al. showed that low Wnt/β-catenin signaling in CVOs leads to leaky vessels, and dominant, endothelial cell-specific β-catenin expression in mice transformed leaky blood vessels into BBB-like vessels by increasing claudin-5 positive vessels, stabilizing junctions and reducing MECA-32 [13]. Notable, there are cross-talks between the MyD88 downstream signaling mediator NFκB and Wnt/β-catenin signaling [14]. The interaction is bidirectional i.e. Wnt/β-catenin can regulate the NFκB activity and NFκB can regulate Wnt/β-catenin signaling, potentially integrating the two pathways in regulating BBB function. In terms of the NFκB effect on Wnt/β-catenin signaling, many studies observe a negative regulation of NFκB on Wnt/β-catenin signaling [14], suggesting that the lack of MyD88 and thereby NFκB signaling in our study could lead to increased Wnt/β-catenin signaling and less leaky blood vessels. Notable, a recently published article showed that intestinal inflammation, leading to gut vascular barrier opening, modulates the brain choroid plexus through bacteria-derived LPS which in turn leads to closure of the brain choroid plexus by the up-regulation of the Wnt/β-catenin signaling pathway, protecting the brain from inflammation [15]. The authors observed a temporal effect of intestinal inflammation, with an early increased permeability in choroid plexus, followed by closure of the choroid plexus [15]. These results could indicate a negative feedback mechanism of LPS via MyD88, that further cross-talk with Wnt/β-catenin signaling.

In contrast to previous studies, showing an effect of the gut microbiota on the BBB [9, 30], we did not observe such effects. Several factors could explain these differences. First, this study focuses on an area of the brain with an incomplete BBB, while previous studies focus on areas with a complete BBB. Different mechanisms are most likely involved in the BBB regulation in these areas, supported by the higher TLR4 expression in the CVOs, and the presence or absence of a gut microbiota may therefore lead to different effects in these areas. Second, gut microbial composition in CONV-R mice from different animal facilities have been shown to differ significantly and can lead to different phenotypes [31, 32]. Thus, it is possible that differences in the CONV-R gut microbial composition can explain differences in the phenotype. As discussed above, the diet is another factor that may affect the results. Different antibiotic protocols have also been used. We used a relatively mild antibiotic treatment compared to previous studies [30]. Our protocol was used due to its minimal effects on the mice feeding behavior. Even though such antibiotic treatment does not completely deplete the gut microbiota, we have previously shown that this antibiotic treatment protocol results in a dramatic depletion of the gut microbiota as well as microbial-produced metabolites [7]. However, differences in antibiotic treatment could potentially cause differences in the phenotype observed both by affecting the gut microbial composition as well as affecting feeding behavior. Antibiotics may also exert intrinsic off-target effects that are not related to the depletion of the microbiota. Taken together, further studies are needed to disentangle how these different factors modulate the effects of the gut microbiota in these studies.

Our study has limitations. First, it should be noted that the microbiota composition was not analyzed after the colonization procedure in this study, and the exact microbial composition in the colonized mice is therefore not known. However, previous studies using a similar protocol, have shown that transplantation of cecal content from one mouse to another mouse results in microbial composition comparable to the donor mouse [33]. Another limitation of the present study is that only female GF mice were used and compared to female CONV-R mice. Thus, sex differences could not be detected. However, in contrast to the GF mice, both male and female mice were included when comparing Myd88 deficient mice to WT mice. In this experiment, female and male mice showed similar phenotype compared to their controls.

In conclusion, our results suggest that MyD88 signaling in CVOs plays a role in the CVOs’ function by decreasing expression of tight junction proteins and thereby allowing neurons to sense blood-borne substances as well as secrete neurohormones to the blood stream. In contrast, our results suggest that modulation of the gut microbiota is not sufficient to alter the BBB function in the mediobasal hypothalamus. Further studies are needed to determine if increased MyD88 signaling in MBH during systemic infection is involved in a negative feedback mechanism leading to closure of the CVO.

## Supplementary Information

Below is the link to the electronic supplementary material.

Supplemental Figure 1. Hypothalamic astrocytes and tanycytes express TLR4. a Representative picture of hypothalamic TLR4 expression in WT CONV-R mice. Scale bars: 100µm. b–c Co-localization of GFAP and TLR4 in CONV-R (n=9) and GF (n=5) WT female mice. Scale bars: 50µm. d–e Co-localization of Vimentin and TLR4 in CONV-R (n=9) and GF (n=5) WT female mice. Scale bars: 100µm. Graphs show mean ± SEM. (PNG 2.29 MB)

High Resolution Image (TIF 23.7 MB)

## Data Availability

The datasets generated during and/or analyzed during the current study are available from the corresponding author on reasonable request.
