# Supplementary figures and images for: MyD88 Deficiency, but Not Gut Microbiota Depletion, Is Sufficient to Modulate the Blood–Brain Barrier Function in the Mediobasal Hypothalamus
Source: Mol Neurobiol. 2022 Apr 6;59(6):3755–66. doi: 10.1007/s12035-022-02802-w (PMC9148286; doi:10.1007/s12035-022-02802-w)

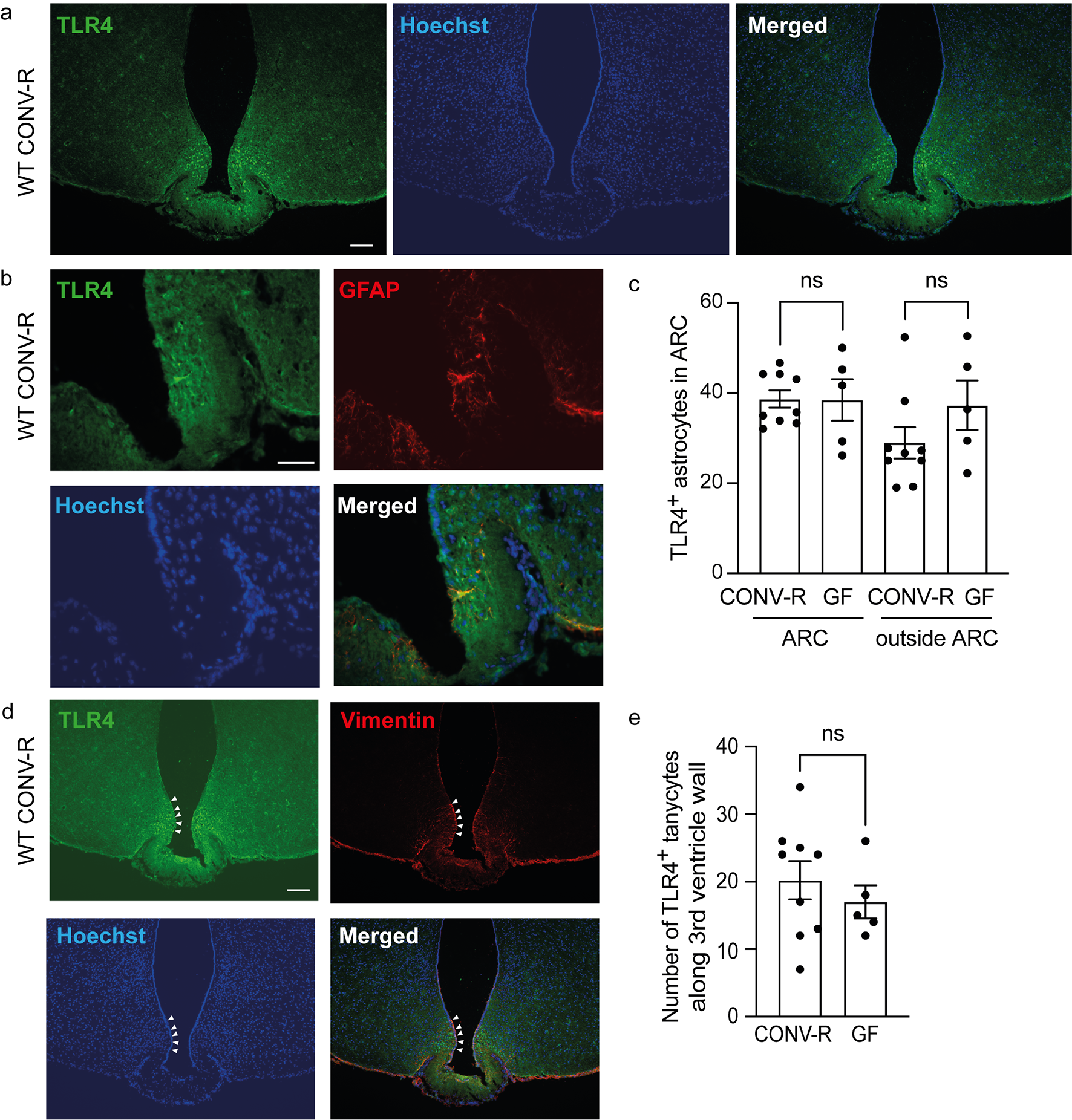

Supplement: Supplementary file 1 — Hypothalamic astrocytes and tanycytes express TLR4. a Representative picture of hypothalamic TLR4 expression in WT CONV-R mice. Scale bars: 100µm. b–c Co-localization of GFAP and TLR4 in CONV-R (n=9) and GF (n=5) WT female mice. Scale bars: 50µm. d–e Co-localization of Vimentin and TLR4 in CONV-R (n=9) and GF (n=5) WT female mice. Scale bars: 100µm. Graphs show mean ± SEM. (PNG 2.29 MB) [file 12035_2022_2802_Fig6_ESM.png]

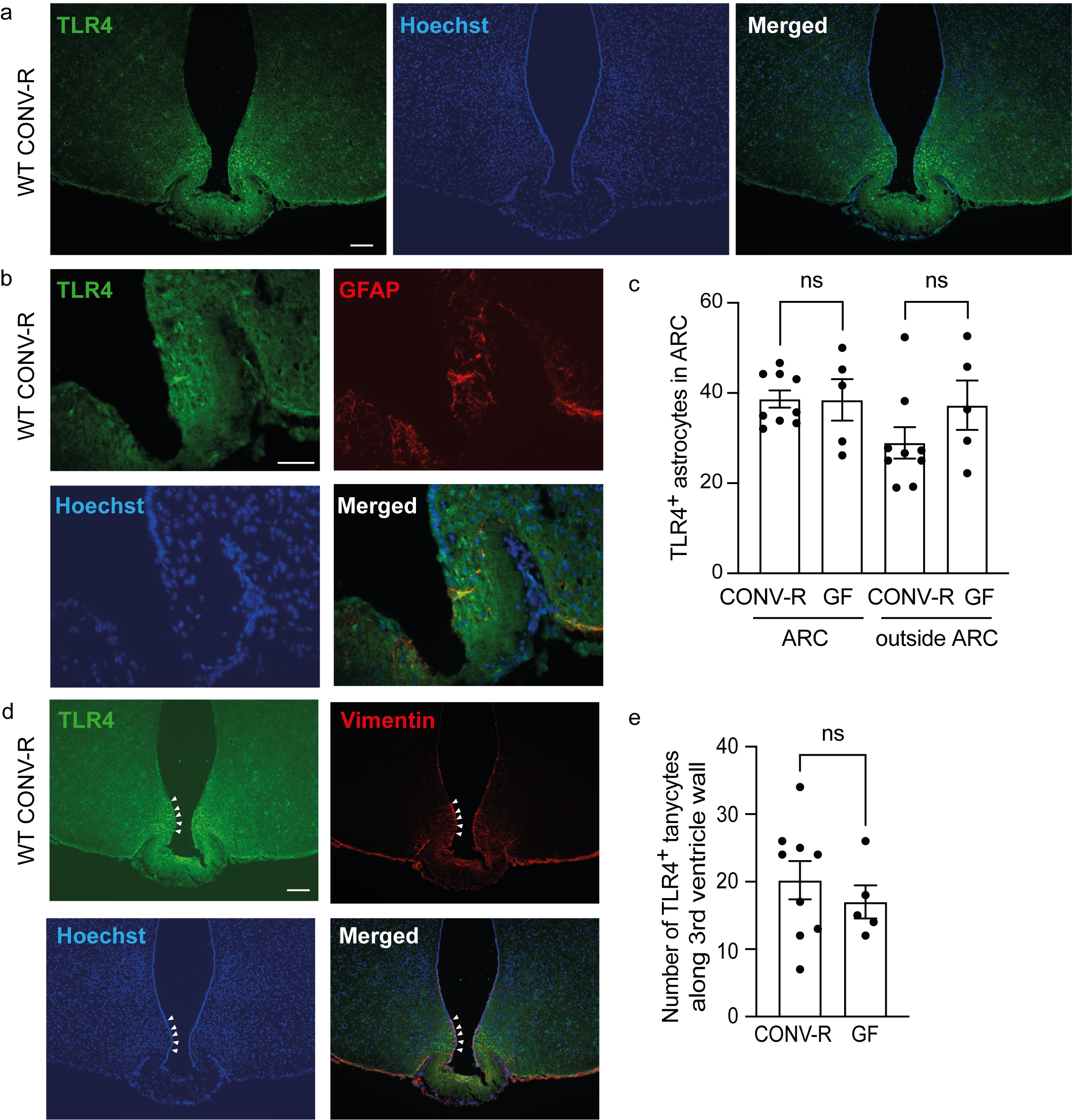

Supplement: Supplementary file 2 — (TIF 23.7 MB) [file 12035_2022_2802_MOESM1_ESM.tif]
